# Research on the Cascade-Connected Transducer with Multi-Segment Used in the Acoustic Telemetry System while Drilling

**DOI:** 10.3390/mi10100712

**Published:** 2019-10-21

**Authors:** Duo Teng

**Affiliations:** 1 School of Marine Science and Technology, Northwestern Polytechnical University, Xi’an 710072, China; tengduo@nwpu.edu.cn; Tel.: +86-29-8849-2599; 2 National Key Laboratory for Underwater Information Processing and Control, Xi’an 710072, China

**Keywords:** cascade-connected transducer, low frequency, small size, finite element, acoustic telemetry, measurement while drilling

## Abstract

The electroacoustic transducer with the performances of low frequency, small size, and high power is desired in the application of the acoustic telemetry system while drilling. In order to fulfill the severe requirements, a novel cascade-connected transducer with multi-segment is developed. The essential framework of such a transducer is to add the cross-beams in the multi-segment cascade-connected arrangement, based on the fundamental configuration of the longitudinal transducer. The flexural vibrations of cross-beams help the transducer to present the appropriate coupling between longitudinal and flexural vibrations, which provide many benefits in keeping the advantages of the longitudinal transducer and lowering the resonance frequency. It is the finite element method to be used for simulating the mode shapes of the cascade-connected transducer, especially the behavior of the cross-beams, and some performances of transducer are also predicted. Several prototypes of cascade-connected transducers with different segments are manufactured. Their related tests show a good agreement with the finite element simulations and analyses. Their characteristics of low frequency, small size, light weight, and high power are attractive for the transmitting or receiving application in the acoustic telemetry system while drilling.

## 1. Introduction

During the drilling operations for exploration and development of oil and gas, the measurements play an important role in obtaining the physical parameters of geologic formations and the monitoring of downhole conditions [1]. This valuable information can be derived in different ways. For example, the drill bit can be withdrawn from the borehole and a “wireline logging tool” can be lowered into the borehole to take measurements [2]. Obviously, such a wireline logging after drilling will not be advantageous in obtaining the real-time and reliability data, as well as in the convenient operation of measurements and drilling [3]. Alternatively, the approach called measurement while drilling (MWD) or logging while drilling (LWD) with real-time data transmission increasingly attracts the attention in the oil and gas industry [4].

MWD is a technology of obtaining real-time measurement of various data at the bottom of the well during the drilling process [5]. Compared with conventional wireline logging, MWD is helpful to avoid the shortages in application [6]. This benefits from the measurement without stopping drilling. In the process of MWD, the communication and transmission between a downhole drilling assembly and a surface of a well are the key technology. A wireless system for telemetering data from downhole to surface will be an effective solution. The available approaches include utilizing such as mud pressure pulse, insulated conductor, electromagnetic wave, or acoustic wave [7]. For example, an acoustic telemetry system (ATS) by means of acoustic waves propagating along drill strings was commercialized in 2000 [8,9]. This demonstrates the value of acoustic telemetry. In ATS, the electroacoustic transducer is an indispensable apparatus. Its performances play a determinative role in transmitting or receiving the acoustic waves, and its further impact may be related to the effect of acoustic telemetry [10]. Obviously, the importance of the transducer cannot be ignored.

In the practical applications, considering the special function and severe downhole situation [11,12], the stringent demands for transducers are derived from confined space to assemble in drill collar, adaptability to acoustic channel along drill strings, high downhole pressure and temperature, strong shock and vibration, etc. These limitations facilitate the transducers to implement the performances of small size, light weight, low frequency, high power, pressure-resisting, heat-resisting, reliability, etc. 

However, the acoustic telemetry transducers that fulfill all the requirements are difficult to develop. There have been some types of transducers to be developed for ATS while drilling, since the use of telemetry by acoustic waves through the drill strings was suggested in the 1940s due to steel tubular such as drill pipe with good and effective acoustic propagation [13]. In 1961, a magnetostrictive cylinder was invented by Woodworth for generating and/or receiving acoustic waves, and it had a low impedance of about 4000 ohms at the frequencies of 10 to 20 kHz [14]. In 1982, an acoustic well-logging transmitting and receiving transducer was invented by Dennis, which comprised the stacked piezoceramic rings and a resonating metallic plate, and operated at a desirable frequency lower than 15 kHz [15]. In 1989, a rare earth acoustic transducer was utilized by Liu to provide low-frequency acoustic energy in an acoustic well logging apparatus. That was attributed to the low acoustic velocity of the Terfenol-D alloys, so it was possible to produce a lower frequency below 10 kHz without increasing the transducer length [16]. In 1997, one acoustic transducer using PZT-8 type piezoelectric ceramic was provided by Drumheller for use in an acoustic telemetry system. The transducer was capable of delivering 10 W of acoustic energy at the frequency of 1.2 kHz, and its efficiency of energy conversion was approximately 40% [17]. In 2015, a transducer comprised of piezoelectric ceramic rings and thin metallic electrodes was tested in the drill strings for the acoustic telemetry by Zhao. The transducer had the performances of broad bandwidth and low frequency below 3.1 kHz [18].

**Figure 1:**
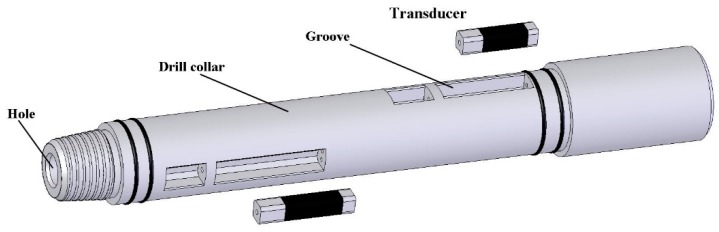
The schematic diagram of a drill collar with a groove to assemble the transducer.

In addition, there are some other types of transducers to be used in ATS. Their different configurations and characteristics were present in references [6,19,20,21,22,23,24]. All the studies provide good guidance. In respect of function realization, many of them can be used as a low-frequency acoustic source. While in respect of special requirements of ATS, some types of transducers still have much room for improvement, especially in miniaturizing the transducer to assemble in drill collar. As a practical application, for the most common drill pipe with a diameter of 178 mm, the maximum transverse size will be only about 40 mm to be provided for the electroacoustic transducer. Figure 1 illustrates the limitations subject to a drill collar of 178 mm in diameter. Obviously, it is difficult to develop the low frequency, high power acoustic source in such a restricted size. 

Many types of transducer, such as Tonpilz transducers, flextensional transducers, flexural transducers, Piezoelectric rings, magnetostrictive transducers, piezoelectric ultrasonic transducers [25,26], are not suitable to MWD application because of the requirements of configurations, vibration modes, radiation field, acoustic impedance matching layers, etc. [27,28]. Under these restrictions of MWD application, the available types of transducer used in an acoustic telemetry system while drilling are few. If just considered as a receiver, MEMS may be an available alternative for detecting the acoustic signals propagated in drill strings. While as a transmitter with high power, the Tonpilz transducer is comparatively suitable because of its longitudinal vibration mode [29]. Generally, Tonpilz transducer can work effectively in the frequency range above 10 kHz. According to the advantages of the acoustic channel in drill strings, low frequency of the transducer will be desired in order to achieve a long-distance transmission, especially below 10 kHz and even lower [30]. In order to overcome the limitations in the ATS application, a type of novel longitudinal-flexural complex-mode low-frequency transducers will be presented.

## 2. Cascade-Connected Transducer with Coupled Longitudinal and Flexural Vibrations

The novel type of low-frequency piezoelectric transducer is called “cascade-connected transducer”, which couples the longitudinal and flexural vibrations. Its longitudinal vibration mode is along the direction of drill strings, which is just in accordance with drill collar. Therefore, the acoustic energy can be transmitted forward along drill strings in the close match between the radiating head of the transducer and the drill collar. This characteristic will offer possibilities to take full advantage of the acoustic channel in drill strings. Its flexural vibration mode plays an important role in lowering the resonance frequency of the transducer. Hence, the above coupling makes it possible to implement the characteristics of low frequency, miniaturization, and high power.

**Figure 2:**
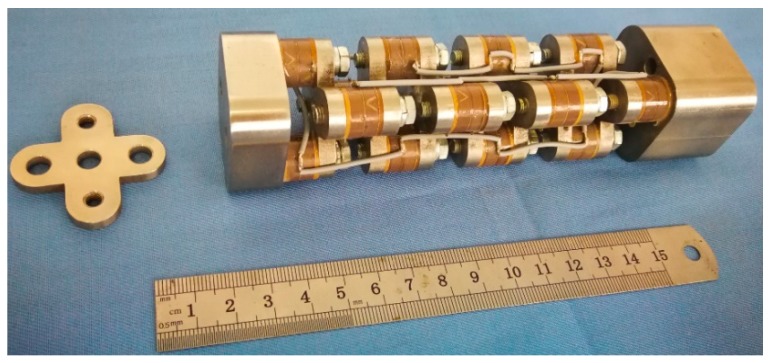
The cascade-connected transducer with eight segments.

The essential framework of the cascade-connected transducer is the multi-segment cascade-connected arrangement connected by the cross-beams, based on the conventional longitudinal transducer. The roles of the beam lie in not only its connection purpose as a structural part but also its flexural vibration as a functional component. Therefore, the Tonpilz configuration is fundamental, the multi-segment cascade-connected arrangement is essential, while the bending beam is the key. Figure 2 illustrates the configuration of the cascade-connected transducer. Its main components include piezoelectric stacks, bending beams, radiating head, tail mass, prestressed bolts, and other appurtenances. The radiating head is the region of transmitting the acoustic energy. Generally, its material should be similar to the drill collar, because the more approximate the characteristic impedances of materials are, the more smoothly the acoustic energy will propagate [31]. Typically, the tail mass should be comparatively heavy metal. A large tail-to-head mass ratio is desirable because it will yield a large head velocity. As the derivation, the more acoustic energy will be transmitted from the head. Every piezoelectric stack is glued closely together in series and wired in parallel. The configuration of multi-segment in a cascade-connected arrangement will be designed to match the length of the groove in the drill collar. There are eight segments in Figure 2. Every segment includes two columns of piezoelectric stacks, fastened to the bending beams alongside each other by a prestressed bolt. A type of cross-beam will be accepted to ensure the cross-connection between the adjacent segments. Compared with the other types of bending beams, this type of cross-beam will provide sufficient benefits to optimize the performances of the cascade-connected transducer, especially to lower the resonance frequency.

## 3. Finite Element Analysis of the Cascade-Connected Transducer 

The models and methods used in transducer analysis and design are always desired. However, it is difficult to develop an ideal solution because the piezoelectric transducer is an integrated system, which needs to be described in three different domains. A piezoelectric transducer is part acoustical at its moving surface in contact with the acoustic medium, part mechanical as a moving body controlled by forces, and part electrical as a current controlled by voltage [32]. So far, the equivalent circuit method (ECM) and finite element method (FEM) are comparatively comprehensive and effective, especially FEM is the prevailing method in the engineering development of transducer.

As a numerical method, the mathematical fundamentals of FEM are variational principle, subdivision, and interpolation [33]. After the whole piezoelectric transducer system is divided into finite elements connected at nodes, the matrix equations of the whole system will be formed. The key to these matrix equations are the governing equations, which can describe the behavior of piezoelectric coupled field. The computer solution of the whole system will be obtained, and the response of any position in the system will be calculated by interpolating. Then, a comprehensive explanation of how the system acts as a whole will be provided. Generally, FEM can model a complicated transducer without large-scale assumptions [34]. The other advantages of convenient modeling, rapid solution, accuracy result, and intuitive illustration are also attractive. Anyway, FEM has become one of the most effective methods to design or simulate the piezoelecteic transducers, especially for the sophisticated configurations, or the complex boundary conditions.

For the piezoelectric finite elements of the transducer model, their inclusion is the governing equation, which can describe the electric structure coupled field problem, written as [35]

(1) $$\left[\begin{array}{cc} M & 0\\ 0 & 0 \end{array}\right]\cdot \left[\begin{array}{c} \ddot{\xi }\\ \ddot{U} \end{array}\right]+\left[\begin{array}{cc} C & 0\\ 0 & 0 \end{array}\right]\cdot \left[\begin{array}{c} \dot{\xi }\\ \dot{U} \end{array}\right]+\left[\begin{array}{cc} K & -K^{Z}\\ K^{Z} & K^{d} \end{array}\right]\cdot \left[\begin{array}{c} \xi \\ U \end{array}\right]=\left[\begin{array}{c} F\\ q \end{array}\right]$$

where [**ξ**] is vector of nodal displacements, [**U**] is the vector of nodal electrical potential, [**M**] is the mass matrix, [**C**] is the damping matrix, [**K**] is the stiffness matrix, [**K^Z^**] is the piezoelectric coupling matrix, [**K^d^**] is the dielectric conductivity matrix, [**F**] is the nodal force vector, [**q**] is the electrical load vector.

For the finite element program, the complete material properties should be specified. Table 1 lists the details as follows.

During the typical process of FEM solution, it is the most important step to build the finite element model. In some ways, the closer to the transducer prototype the model is, the more accurate the solution will be. However, in practice, in order to reduce the modeling difficulties or save the calculation time, simplifying the model without influencing the accuracy of the solution will be helpful. For our cascade-connected transducer, a 1/4 symmetrical finite element model will be built according to some assumptions. The following will present two results of finite element analysis, based on two different configurations of cascade-connected transducers including twelve segments and ten segments respectively.

### 3.1. Cascade-Connected Transducer with Twelve Segments

**Figure 3:**
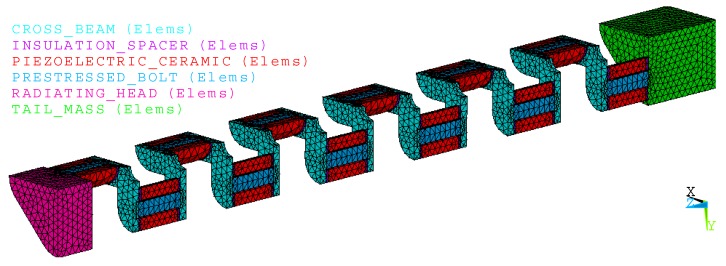
Finite element model of cascade-connected transducers with twelve segments.

Figure 3 illustrates the finite element model of the cascade-connected transducer, which is symmetrical to the XOZ and YOZ plane. The model includes 45,499 nodes and 22,519 elements. The different colors show the different components of the transducer. Its detailed sizes are as follows. The radiating head is 38 × 38 × 15 mm, the tail mass is 38 × 38 × 30 mm, the thickness of cross-beam is 5 mm and the piezoelectric ceramic ring is Φ 14 × 4 mm with a hole of Φ 6 mm. The cascade-connected transducer includes twelve segments, every segment includes two columns of piezoelectric stacks, and every piezoelectric stack includes four piezoelectric ceramic rings.

**Figure 4:**
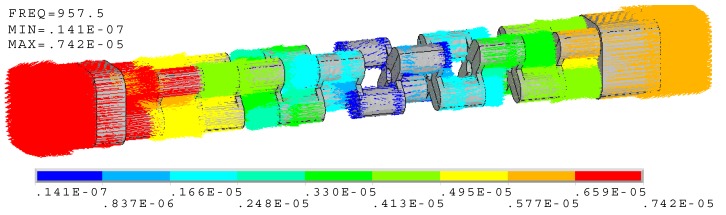
Vector illustration of vibration at 957.5 Hz (cascade-connected transducer with twelve segments).

When the finite element model is assumed to be free at both the head and tail end, Figure 4 illustrates the mode of vibration. The modal frequency is near 957 Hz, which is the resonance frequency of the cascade-connected transducer with twelve segments. On the whole viewpoint, the mode shape is longitudinal. Essentially, the piezoelectric stacks, the head, the tail, and the bolts are all longitudinal, while the cross-beams are flexural (their mode shape will be shown in Section 4). The obvious vibrations occur at the ends of the transducer, and comparatively the radiating head section is stronger than the tail mass section. This indicates that more energy is transmitted from the head. 

**Figure 5:**
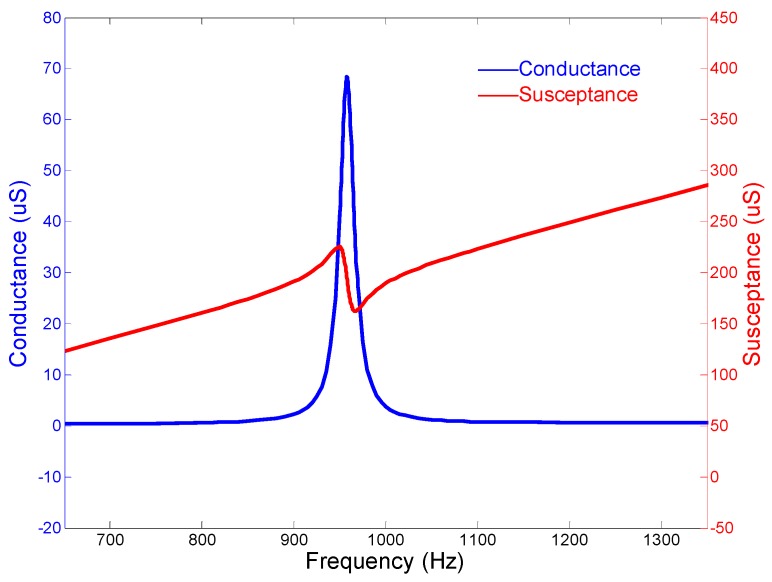
Admittance curves of the cascade-connected transducer with twelve segments.

Figure 5 presents the admittance performances of the cascade-connected transducer. The curves show that the peak of the conductance curve appears at 957.5 Hz, which is near the resonance frequency derived from the coupled longitudinal and flexural vibrations. The sharp peak of the conductance curve also predicts that the bandwidth of the cascade-connected transducer is narrow. Therefore, this type of transducer cannot be used as a broadband transducer individually.

### 3.2. Cascade-Connected Transducers with Ten Segments

**Figure 6:**
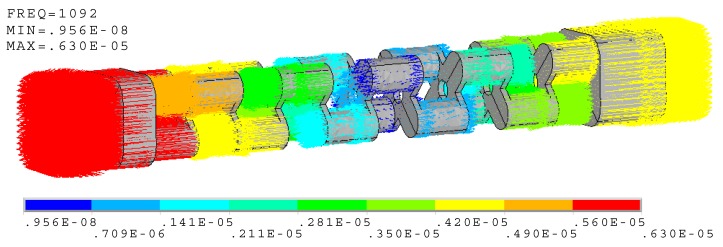
Vector illustration of vibration at 1092 Hz (cascade-connected transducer with ten segments).

**Figure 7:**
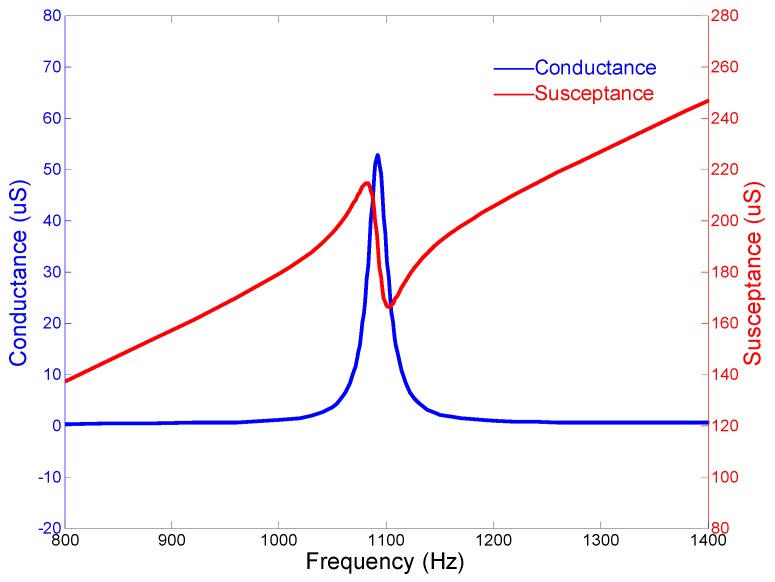
Admittance curves of the cascade-connected transducer with ten segments.

A similar cascade-connected transducer, which includes ten segments, is also presented. All the configurations are the same except the number of segments. The finite element model of the cascade-connected transducer with ten segments is the same as the above. The results are also similar. Figure 6 shows the mode of vibration and Figure 7 shows the admittance curves. They predict that the resonance frequency is near 1092 Hz. 

## 4. Cross-Beams

The bending beams are one of the main differences between the cascade-connected transducer and the conventional Tonpilz transducer. The beams should be helpful for enhancing the longitudinal vibration, and also for lowering the resonance frequency of the transducer. Therefore, the bending beams need to be designed and optimized. The cross-beam is a good alternative. The material and the thickness of the beam are the major factors to control the flexural mode of cross-beam. The flexural mode, as an indispensable vibration of the whole, plays an important role in the cascade-connected transducer. Hence, its performances need to be comprehended.

**Figure 8:**
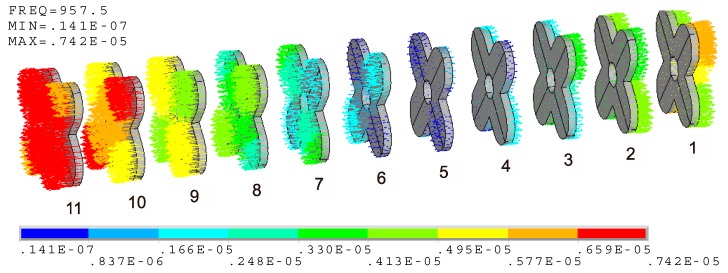
Vector illustration of vibration at 957.5 Hz (cross-beams).

For the cascade-connected transducer with twelve segments, there need to be eleven cross-beams. The serial number of each beam is shown in Figure 8, where the beam near the tail is assigned as No. 1 and the beam near the head is assigned as No. 11. According to the above analysis, a vector illustration of vibration of cross-beams is shown in Figure 8, which is separated from the whole system in Figure 4. The result shows that the move of the cross-beams near the ends of the transducer is significant, while the move of the middle cross-beam is low. The vibrations of both ends are opposite. All of the performances agree with the longitudinal vibration of the cascade-connected transducer. Although the longitudinal move of the cross-beam is real, it disguises the fact of flexural vibration. The reason is that each cross-beam is attached to a constant move of longitudinal vibration, which is provided by the whole system. This constant longitudinal move of each beam is different, but every one is larger than the scope of its other vibrations. If the constant longitudinal move of each beam is separated, only the flexural vibration will be left. 

**Figure 9:**
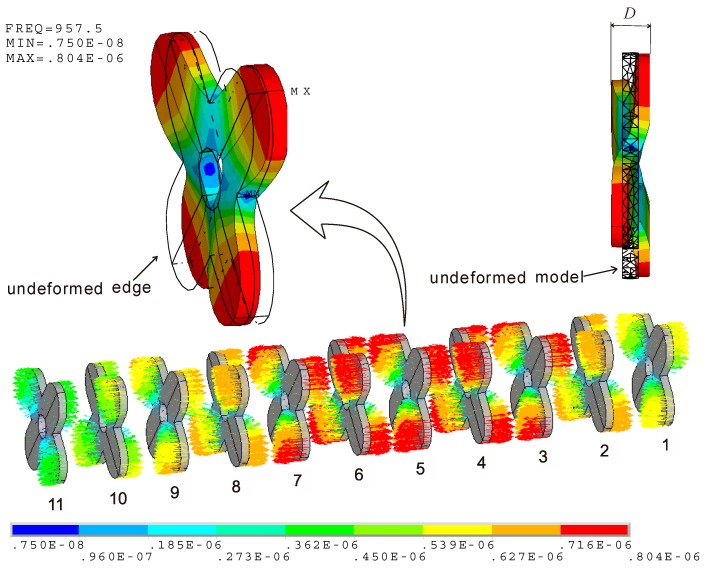
Vector illustration of flexural vibration at 957.5 Hz (cross-beams).

Figure 9 illustrates that the clear flexural vibration happens to each cross-beam. Their distinctions are just the vibration intensity. Unlike the before-mentioned longitudinal move of the cross-beams, the flexural vibrations of the cross-beams near the ends of the transducer are weak, while those of the middle cross-beam are strong. The strongest flexural vibration occurs at No. 5 beam. Figure 9 illustrates its mode shape. If the undeformed edge is assigned as the reference, it is clear that the two orthogonal branches of the cross-beam vibrate respectively along opposite directions. This mode shape is propitious to enhance the longitudinal vibration of the cascade-connected transducer, and also to achieve the low resonance frequency. This is the advantage of cross-beam. 

**Figure 10:**
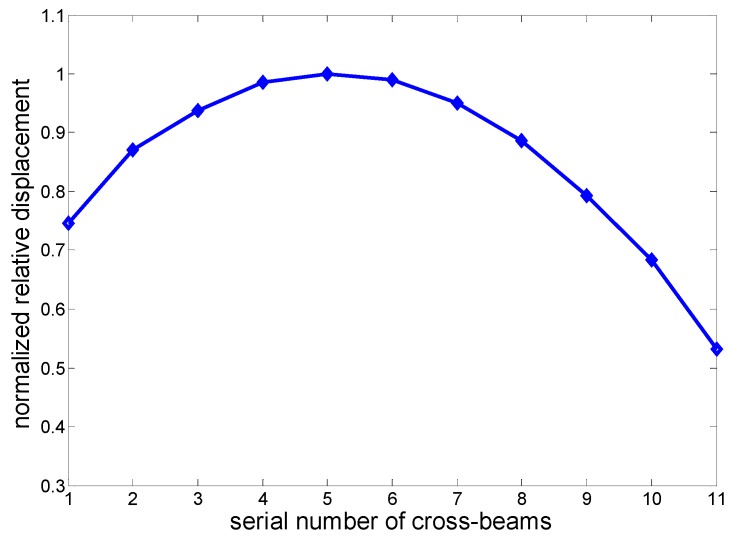
Normalized relative displacements of all the cross-beams.

If the maximum relative displacement between the two orthogonal branches of each cross-beam is defined as *D_i_*, shown in Figure 9, and the subscript “*_i_*” stands for the serial number of each cross-beam, the value of *D*_5_ is maximum. Figure 10 illustrates the relative displacements of all the cross-beams. Its *Y*-axis label is the normalized relative displacement $\frac{D_{i}}{D_{5}}$, and its *X*-axis is the serial number of beams. *D_i_* of the beams at the two ends are dissymmetric because the masses of the head and tail are different.

## 5. Test and Discussion

**Figure 11:**
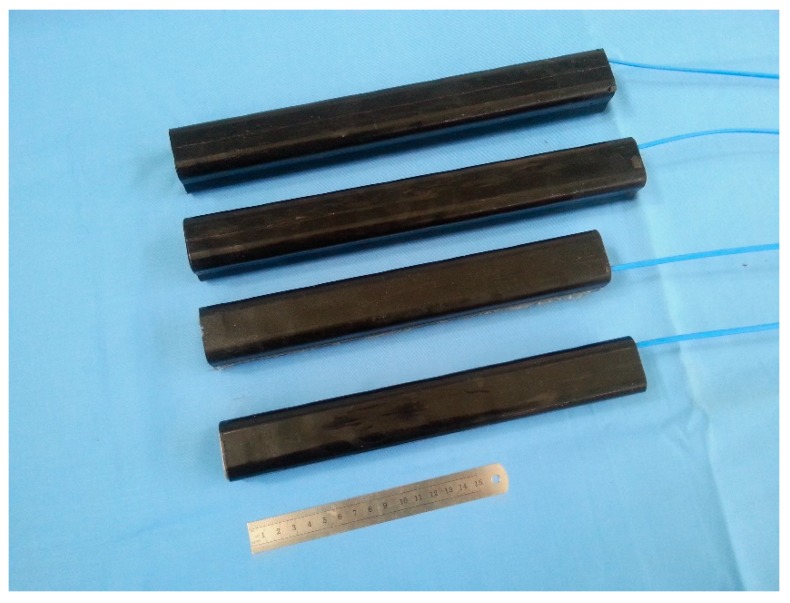
Prototypes of cascade-connected transducers.

Some cascade-connected transducer prototypes have been manufactured according to the above designs. Two kinds of cascade-connected transducer have the external size of 38 × 38 mm. The prototype with twelve segments is 310 mm long and weighs 1.23 kg, while the one with ten segments is 266 mm long and weighs 0.88 kg. Figure 11 shows the prototypes of cascade-connected transducers, which are encapsulated in polyurethane rubber.

**Figure 12:**
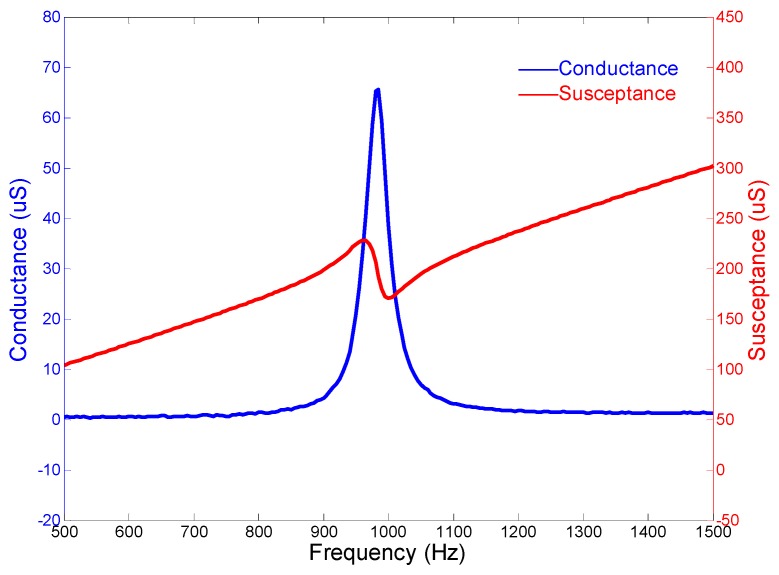
Admittance curves of cascade-connected transducers with twelve segments (obtained from Agilent 4294A).

**Figure 13:**
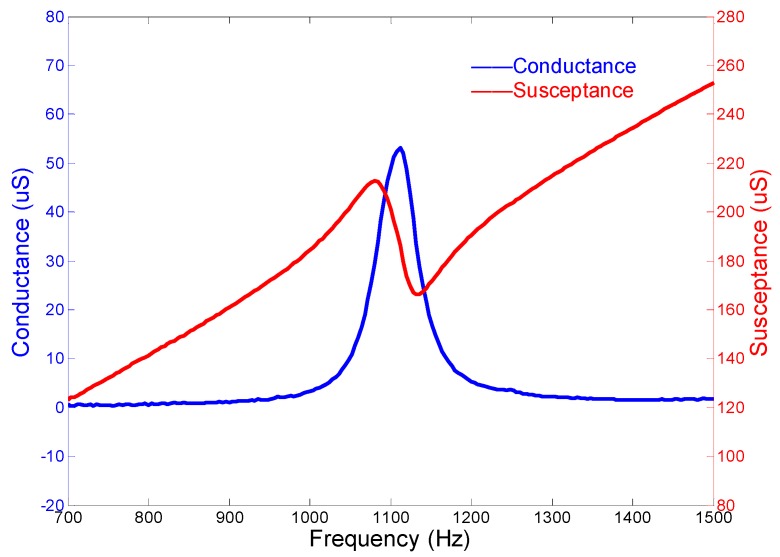
Admittance curves of cascade-connected transducers with ten segments (obtained from Agilent 4294A).

**Figure 14:**
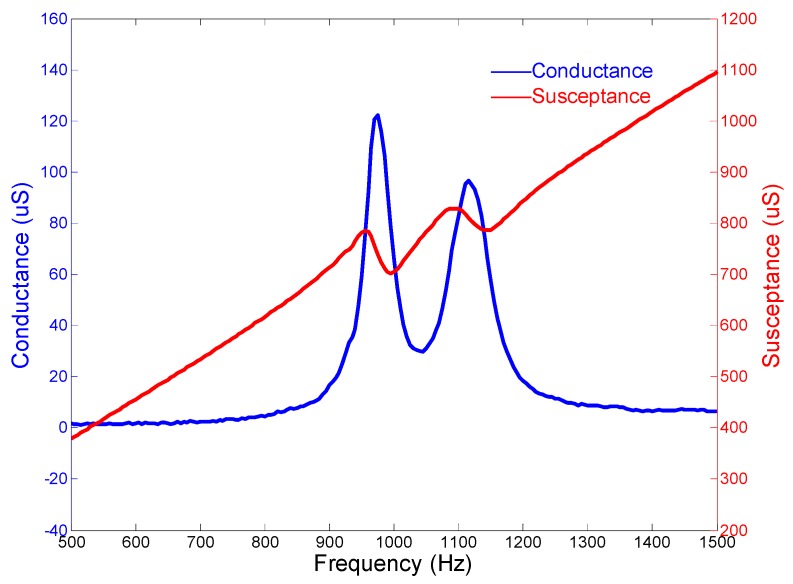
Admittance curves of four wired cascade-connected transducers (two for twelve segments and two for ten segments).

The admittance curves are obtained from the precision impedance analyzer Agilent 4294A (Agilent, Santa Clara, CA, USA). Figure 12 shows the curves of the cascade-connected transducer with twelve segments. The curves show that the resonance frequency is near 985 Hz, which is slightly higher than the result of FEM. The admittance curves of the cascade-connected transducer with ten segments are shown in Figure 13. Its resonance frequency is near 1112 Hz. The comparison between the measurements and the simulations illustrated respectively in Figure 5 or Figure 7 shows consistency. In practice, the two kinds of cascade-connected transducers can be wired together in parallel to broaden the bandwidth. Figure 14 illustrates the admittance curves of four wired cascade-connected transducers (two for twelve segments and two for ten segments, shown in Figure 11). There are two adjacent peaks. Their coupling can optimize the performances of transducers, which will provide higher power and wider bandwidth to achieve a better effect in the acoustic telemetry system while drilling.

Based on the measurements of resonance frequency, *f_r_*, and anti-resonance frequency, *f_a_*, the transducer’s effective electromechanical coupling coefficient, *k_eff_*, can be calculated. The two kinds of cascade-connected transducer have *k_eff_* of 10.11% and 11.93%, respectively.

For a transmitting transducer, the performance of high power is always desired. The power consumption of two kinds of cascade-connected transducers is monitored from a small excitation signal to high driving voltage. The increasing response is basically linear. A safe operating state is as follows. The power consumption of single cascade-connected transducer with twelve segments is 30.9 watts at the frequency of 970 Hz, where the driving voltage is 1188 V p–p (peak to peak), the current is 243 mA p–p, and the current leads the voltage by 64.6 degrees. The power consumption of a single cascade-connected transducer with ten segments is 31.3 Watts at the frequency of 1110 Hz, where the driving voltage is 1188 V p–p, the current is 234 mA p–p, and the current leads the voltage by 63.2 degrees. These is not the maximum power consumption for the transducers because of the limitation of the power amplifier.

## 6. Conclusions

A novel type of cascade-connected transducer is present. Its performances are predicted through finite element method, especially the behavior of cross-beams. Several prototypes with different segments are manufactured. The relevant tests show that the cascade-connected transducer can satisfy the requirements for the transmitting or receiving application in the acoustic telemetry system while drilling. In summary, the following conclusions can be obtained.

1. The major configuration of the proposed transducer is the multi-segment cascade-connected arrangement connected by the cross-beams, based on the conventional longitudinal transducer. Such design not only keeps the advantages of longitudinal radiation and high power, but also provides the characteristics of low frequency and small size.
2. The cascade-connected transducer utilizes longitudinal and flexural complex mode. The coupling of two vibration modes is the key to achieve the desired electroacoustic performances. During this process, the cross-beam plays a pivotal role based on its flexural vibration. Therefore, the design and optimization of the bending beams is an important research point.
3. The simulation and test show that the cascade-connected transducer has a resonance frequency of approximately 1 kHz. If the number of segments in the cascade-connected transducer increases, the operating frequency will be lowered. Hence, besides the bending beam, the number of segments is also the deciding factor to lower the resonance frequency.
4. The simulation based on the finite element method shows that cascade-connected transducer can lower the resonance frequency by approximately 80% more than the conventional Tonpilz type transducer when the two transducers have the same length.
5. The relevant tests show that the effective electromechanical coupling coefficient, *k_eff_*, of cascade-connected transducer is approximately 11%. It is less than the value of conventional Tonpilz type transducer. There are many factors which can affect *k_eff_* in practice. One possible reason is that the small radiation surface leads to the small radiation resistance at low frequency.

## Figures and Tables

**Table 1:** Material properties of cascade-connected transducer.

Components	Material	Quantity	Material Property [32]
Piezoelectric stack	PZT-4 piezoelectric ceramic	Density (kg/m^3^)	7600
		Stiffness coefficients matrix (×10^10^ N/m^2^)	$c^{E}=\left[\begin{array}{llllll} 13.9 & 7.78 & 7.43 & 0 & 0 & 0\\ 7.78 & 13.9 & 7.43 & 0 & 0 & 0\\ 7.43 & 7.43 & 11.5 & 0 & 0 & 0\\ 0 & 0 & 0 & 3.06 & 0 & 0\\ 0 & 0 & 0 & 0 & 2.56 & 0\\ 0 & 0 & 0 & 0 & 0 & 2.56 \end{array}\right]$
		Piezoelectric stress matrix (C/m^2^)	$e=\left[\begin{array}{lll} 0 & 0 & -5.2\\ 0 & 0 & -5.2\\ 0 & 0 & 15.1\\ 0 & 0 & 0\\ 0 & 12.7 & 0\\ 12.7 & 0 & 0 \end{array}\right]$
		Relative permittivity matrix	$\frac{\varepsilon^{S}}{\varepsilon_{0}}=\left[\begin{array}{lll} 730 & 0 & 0\\ 0 & 730 & 0\\ 0 & 0 & 635 \end{array}\right]$
Radiating head Tail mass Cross-beam	Steel	Density (kg/m^3^) Young’s modulus (N/m^2^) Poisson’s ratio	7840 2.16 × 10^11^ 0.28

## References

[1] Gravley W. (1983). Review of downhole measurement-while-drilling systems. J. Pet. Technol..

[2] Snyder D.D., Fleming D.B. (1985). Well logging—A 25-year perspective. Geophysics.

[3] Johnson H.M. (1962). A history of well logging. Geophysics.

[4] Su Y.N., Dou X.R. (2005). Measurement while drilling, logging while drilling and logging instrument. Oil Drill. Prod. Technol..

[5] Inglis T.A., Inglis T.A. (1987). Measurement while drilling (MWD). Directional Drilling.

[6] Shah V.V., Linyaev E.J., Kyle D.G., Gardner W.R., Moore J.L. (2008). Acoustic telemetry transceiver. U.S. Patent.

[7] Spies B.R. (1996). Electrical and electromagnetic borehole measurements: A review. Surv. Geophys..

[8] Shah V.V., Gardner W.R., Johnson D.H., Sinanovic S. Design considerations for a new high data rate LWD acoustic telemetry system. Proceedings of the SPE Asia Pacific Oil and Gas Conference and Exhibition.

[9] Gardner W.R., Shah V.V. (2001). High Data Rate Acoustic Telemetry System. U.S. Patent.

[10] Squire W.D., Whitehouse H.J. A new approach to drill-string acoustic telemetry. Proceedings of the SPE Annual Technical Conference and Exhibition.

[11] Drumheller D.S. An overview of acoustic telemetry. Proceedings of the Office of Scientific & Technical Information Technical Reports.

[12] Baggeroer A. (2012). An overview of acoustic telemetry: 2001–2011. J. Acoust. Soc. Am..

[13] Drumheller D.S. (1989). Acoustical properties of drill strings. J. Acoust. Soc. Am..

[14] Woodworth J.H. (1961). Acoustic Logging Transducer. U.S Patent.

[15] Dennis J.R. (1982). Acoustic Well-Logging Transmitting and Receiving Transducers. U.S. Patent.

[16] Liu O.Y. (1989). Acoustic Well Logging Method and Apparatus. U.S. Patent.

[17] Drumheller D.S. (1997). Acoustic Transducer. U.S. Patent.

[18] Zhao G.S., Wang B.B., Du Z.C., Huang M.Q., Zhai W.T. (2015). Experimental study on the transmission characteristics of transducer acoustic signal within drill string. Sci. Technol. Eng..

[19] Kent W.H. (1981). Resonant Acoustic Transducer System for a Well Drilling String. U.S. Patent.

[20] Drumheller D.S. (1993). Electromechanical Transducer for Acoustic Telemetry System. U.S. Patent.

[21] Tochikawa T., Sakai T., Taniguchi R., Shimada T. Acoustic telemetry: The new MWD system. Proceedings of the SPE Annual Technical Conference and Exhibition.

[22] Birchak J.R. (1997). Acoustic Transducer for LWD tool. U.S. Patent.

[23] Drumheller D.S. (2000). Acoustic Transducer. U.S. Patent.

[24] Wisniewski L.T., Arian A., Varsamis G.L. (2001). Transducer for Acoustic Logging. U.S. Patent.

[25] Mo X.P., Zhu H.Q. (2012). Thirty years’ progress of underwater sound projectors in China. AIP Conf. Proc..

[26] Meng X.D., Lin S.Y. (2019). Analysis of a cascaded piezoelectric ultrasonic transducer with three sets of piezoelectric ceramic stacks. Sensors.

[27] Gao L., Gardner W., Robbins C., Memarzadeh M., Johnson D. (2008). Limits on data communication along the drillstring using acoustic waves. SPE Reserv. Eval. Eng..

[28] Qiao W.X., Ju X.D., Che X.H., Lu J.Q. (2011). Progress in acoustic well logging technology. Well Logging Technol..

[29] Massa D.P. An Overview of Electroacoustic Transducers. https://www.massa.com/wp-content/uploads/DPM_Overview_of_Electroacoustic_Transducers.pdf.

[30] Drumheller D.S. (1993). Attenuation of sound waves in drill strings. J. Acoust. Soc. Am..

[31] Buckingham M.J., Neighbors T.H., Bradley D. (2017). Sound Propagation. Applied Underwater Acoustics.

[32] Butler J.L., Sherman C.H. (2016). Transducers and Arrays for Underwater Sound.

[33] Hladky-Hennion A.C., Dubus B. (2008). Finite Element Analysis of Piezoelectric Transducers. Piezoelectric and Acoustic Materials for Transducer Applications.

[34] Wang J.J., Qin L., Li W.J., Song W.B. (2019). Parametric Analysis and Optimization of Radially Layered Cylindrical Piezoceramic/Epoxy Composite Transducers. Micromachines.

[35] Allik H., Hughes T.J.R. (1970). Finite element method for piezoelectric vibration. Int. J. Number Meth. Eng..

